# A transparent and standardized performance measurement platform is needed for on-prescription digital health apps to enable ongoing performance monitoring

**DOI:** 10.1371/journal.pdig.0000656

**Published:** 2024-11-15

**Authors:** Cindy Welzel, Stefanie Brückner, Celia Brightwell, Matthew Fenech, Stephen Gilbert

**Affiliations:** 1 Else Kröner Fresenius Center for Digital Health, TUD Dresden University of Technology; Dresden, Germany; 2 Chair of Digital Cultures, TUD Dresden University of Technology; Dresden, Germany; 3 Una Health GmbH, Hamburg, Germany; Iran University of Medical Sciences, ISLAMIC REPUBLIC OF IRAN

Apps on prescription have been introduced in a number of EU countries, with Germany leading through innovative policies and laws. New policy initiatives have recognized the need for greater transparency on the use and positive healthcare effect of these digital health applications and to closely align their pricing to their performance. Performance monitoring includes the assessment of the efficiency, usability, and safety of health apps. We propose a platform-based, automated, and standardized approach for ongoing real-world data collection and analysis. This would provide data on ongoing performance of comparable apps to stakeholders, including regulators, developers, healthcare providers, and patients enabling performance-based pricing of apps on prescription. Besides delivering transparency, another potential benefit is a reduction in developer effort in creating unique data gathering solutions, allowing them to focus their effort on maximizing the performance of their app. Such an approach would maximize the benefit for the whole digital health sector, by incentivizing progress towards providing ever increasing value for patients and caregivers.

“Apps on prescription” (AOPs), known in Germany as DiGA (Digitale Gesundheitsanwendungen) were introduced in Germany in 2019 [1] becoming available to approximately the 73 million people who are covered by German statutory health insurance. These are apps with approval as medical devices of low-risk class (I, IIa, and recently, since the new digitization law came into force, also IIb), which are intended to support the detection and treatment of disease, as well as to support a self-determined and healthy lifestyle. They can be either used by patients alone or by patients in partnership with their healthcare providers (HCPs). DiGA can be prescribed by physicians or psychotherapists. Alternatively, patients with a confirmed indication for which a DiGA exists can get approval through a direct request to the health insurer [1].

From 1 January 2024, new interoperability requirements came into force and DiGA must enable the regular, automated export of data collected to the patient’s electronic health record (EHR) [2]. In addition, a new digital law (Digital-Gesetz), which was passed at the end of 2023 and came into force on 26 May 2024, includes a number of new requirements for DiGA and their pricing [3]. The new law proposes an ongoing performance measurement for DiGA and by 2026 at the latest, performance-related price components should account for at least 20% of the remuneration amount for DiGA [3]. The law does not clearly define the data and the methods for assessment, but instead enables the German Federal Ministry of Health to further define these points in a separate statutory regulation. The law does however provide a high-level overview of what a performance assessment framework should look like. Based on these new requirements, we propose a platform concept that would, first and foremost, enable ongoing performance measurement and would also facilitate performance-related pricing of AOPs.

## DiGA and AOPs in European and international contexts

Since the first inclusion of DiGA into the DiGA directory in 2020 [4], 451,000 apps were prescribed from which 83% (374,377 apps) were actually downloaded and activated by patients [5]. The German DiGA program has been held up as a model for other countries [6] and a number of other European countries have followed the German lead, launching or planning similar AOP programs. For example, in Belgium the mHealth Pyramid was introduced offering 3 levels that evaluate a CE (Conformité Européenne)-certified app on the basis of risk, interoperability, and clinical evidence [7]. The app can enter the lowest level (M1) if it has a CE-certification received from the Federal Agency for Medicines and Health Products (FAMHP) and can climb the hierarchy to M2 if it fulfills interoperability and connectivity requirements to ensure exchange of data between all healthcare stakeholders. It reaches the third level (M3^-^) when it is in the process of proving its positive healthcare effect and is then temporarily financed by the National Institute for Health and Disability Insurance (NIHDI). Once this proof is successfully completed the AOP reaches the top level (M3^+^) and the reimbursement through NIHDI becomes permanent. France is following the German example more closely and has introduced a procedure similar to the German fast-track procedure. The PECAN (prise en charge anticipée des dispositifs médicaux numériques) system allows health insurance companies to cover the costs for 1 year, during which the AOP developers can generate the conclusive clinical evidence of the positive healthcare effect (apart from the mandatory clinical evaluation in accordance with the MDR). If this positive healthcare effect is proven, the app is then categorized as “important,” “moderate,” or “low.” Following price negotiations with the Social Security Fund Caisse Primaire d’Assurance Maladie (CPAM), the AOPs are then reimbursed accordingly [8,9]. While Germany, Belgium, and France are the leading countries in terms of AOP programs, some other European countries, such as Italy, Sweden, and Austria, are now following suit with the development of their own similar programs [10].

Moreover, the need for suited regulatory, health technology assessment, and reimbursement pathways of health apps was also recognized at an international level, for example in the United States (US) the Food and Drug Administration (FDA) introduced a Software Precertification (Pre-Cert) Pilot Program in order to advance digital health technology approvals. This program is expected to enhance the development of a future regulatory model that provides more streamlined and efficient oversight of software as a medical device, including digital health applications. The reimbursement in the US depends on the classification of the device and the insurance scheme [11,12]. Building on these advancements, recently, the Centers for Medicare and Medicaid Services (CMS) of the US Department of Health and Human Services proposed a plan to reimburse doctors for subscription costs and app fees associated with digital mental health treatments [13]. This proposal aims to cover fees for programs that are used in conjunction with ongoing behavioral healthcare treatment and would be a significant boost for health-tech companies, which have faced challenges partly due to limited insurance coverage options. If approved, the new policy will be implemented in 2025 [14]. This does not include independent usage from persons insured on Medicare because the digital therapy tool needs to be provided incident to or integral to professional behavioral health services. The coverage will be limited, applying exclusively to products approved by the FDA. Additionally, CMS has pledged to monitor the use of these digital tools in behavioral healthcare, allowing for potential adjustments. There is substantial hope that Medicare’s reimbursement proposal could make the businesses of digital health applications more scalable, providing a consumer base that does not need to pay out of pocket [13].

## Requirements for AOPs

What all of the abovementioned European AOP systems have in common is that they have to fulfill general requirements including safety, functionality, and data protection, as well as quality and interoperability (Table 1). In addition, they need to demonstrate their positive healthcare effect before they are reimbursed. This means that they must submit sufficient clinical data to demonstrate that the AOP provides either a medical benefit (e.g., improvement of the health status, shortening of disease duration, prolongation of survival, or improvement of quality of life) or a patient-relevant structural and procedural improvement in care (e.g., better coordination of treatment processes or easier access to care) [15]. This generally requires a randomized controlled trial (RCT) [16] and although this is the gold standard of medical evidence generation, RCTs also have major drawbacks, such as the substantial resources required (time, staff, and money), problems with generalizability (participants that volunteer to participate might not be representative of the population being studied), and loss to follow up [17–19].

**Table 1:** Requirements for AOPs in the 3 leading countries Germany, Belgium, and France.

DiGA (Germany)	mHealth (Belgium)	PECAN (France)
Developer of CE-certified health app (class I, IIa, and soon IIb) submits DiGA application to BfArM	Level M1: Registration of CE-certified health app to FAMHP	Developer of CE-certified health app (class I, IIa, IIb, III) submits application to CNEDIMT and HAS
BfArM examination →general requirements (safety, functionality, data privacy, interoperability) → clinical evidence of positive healthcare effect	Level M2: Assessment by e-health platform → risk assessment (data security, privacy, medical confidentiality) → interoperability and connectivity assessment	Idea of co-design: joint assessment by CNEDIMT, HAS and end users → Medico-technic assessment (general HTA) → Assessment of positive healthcare effect
Fast-Track: clinical evidence underway, within a period of 12 months → preliminary DiGA listing	Level M3^-^: Process of proving positive healthcare effect → temporarily financed by NIHDI	Fast-Track: clinical evidence underway, within a period of 6 to 9 months
Clinical evidence met → permanent DiGA listing → price negotiations with GKV-SV and reimbursement	Level M3: Clinical evidence evaluation → approval by NIHDI → reimbursement	Clinical evidence met → price negotiations and final decision by CPAM and ministry of health

BfArM, Bundesinstitut für Arzneimittel und Medizinprodukte (Federal Institute for Drugs and Medical Devices); GKV-SV, Gesetzliche Krankenversicherung-Spitzenverband (National Association of Statutory Health Insurance Funds); FAMHP, Federal Agency for Medicines and Health Product; NIHDI, National Institute for Health and Disability Insurance; CNEDIMT, Medical device and health technology evaluation committee; HAS, Haute Autorité de santé (French National Authority for Health); CPAM, Social Security Fund Caisse Primaire d’Assurance Maladie.

Focusing on Germany and France, there is the option for the AOP (DiGA, PECAN app) to be provisionally reimbursed in case the evidence generation for proving the AOP’s positive impact on healthcare is not fully completed but underway, when all of the other requirements are fulfilled. The AOP developer can then conduct the necessary comparative study within a trial period of 1 year. During this trial period, the price for the AOP is set by the developer with some constraints, but once the AOP has been permanently included in the directory, the reimbursement amount is negotiated between the developer and the national association of health insurance providers (e.g., GKV Spitzenverband in Germany or CPAM in France), replacing the price set by the developer. If the AOP is included in the directory, the cost is reimbursed by the statutory health insurance funds.

## Do AOPs have a positive healthcare effect?

In order to prove the AOPs positive healthcare effect, AOP developers generally perform RCTs, since this is the gold standard of medical evidence generation [20]. However, in RCTs, apps are assessed in highly controlled settings and on highly selected populations. As a result of recruitment bias and the applied exclusion criteria, the participants may only represent a subset of the user population. Therefore, an important aspect of AOP evaluation is through real-world evidence (RWE) [21]. RWE is generally gathered from real-world settings and on broader and more heterogeneous study groups. Therefore, it has advantages in reflecting the spectrum of actual use scenarios rather than anticipated user populations and use situations. Although both RCTs and RWE have advantages and disadvantages they should ideally be developed and executed in a complementary fashion rather than being seen as competing approaches [22].

We propose a concept for the ongoing evaluation of AOPs, which can also be used to assess other digital health applications, based on standardized real world data collection, evaluation, and communication, which is consistent with the high-level requirements set out in the German digital law and would allow the above objectives to be achieved. The new digital law proposes that AOP developers must regularly transmit anonymized aggregated assessment data to the regulator, who will in turn regularly publish and update the data, in order to ensure more transparency about AOP usage and quality [3]. Data to assess AOP performance could include metrics on the average frequency and duration of use, the average course of use or dropout rates, and surveys on user satisfaction [3]. Our proposal emphasizes the need for appropriate performance assessment systems that are not only limited to study settings but adapted to continuous assessment of real-world scenarios. The aims of the creation of such systems are to increase competition between AOP developers on performance, to create a foundation for real-world performance (RWP)-based compensation of AOPs, and to support patients’ and HCPs’ decision-making processes when choosing an appropriate AOP for treatment. This performance assessment platform would make it possible to integrate performance-related price components into the remuneration amount in accordance with the German digital law on the basis of the data on RWP collected during the 1-year trial period. After this 1-year trial period, the transparent and ongoing performance measurement of the AOP would continue to ensure the performance corresponding to the negotiated remuneration amount.

The use of digital health applications, including AOPs, is increasing not only in Germany but also in Europe and worldwide [5,10,23,24] and the importance of identification of safe and effective apps for patients and HCPs is an issue of ongoing concern at supranational level [11,25]. Major issues include lack of information for patients and HCPs about the quality of AOPs, uncertainties about the optimal process to rapidly assess evidence on the positive healthcare effect, as well as technical concerns [26–32]. The platform approach that we propose addresses these concerns by providing more transparency about AOP performance for patients and HCPs and provides additional evidence from real-world scenarios alongside RCTs to demonstrate the positive healthcare effect, thus providing a foundation for performance-based pricing decisions. Since the basic principles of good evidence generation are similar across different countries, e.g., all AOPs have to demonstrate a positive healthcare effect as well as conformity with safety and functionality requirements, our concept for AOP monitoring is applicable not only to Germany—the same approach could be used in other countries, and, if there was political will, could even enable the comparison of AOPs between different countries.

## A continuous performance monitoring system for AOPs

We bring together existing solutions and already technically feasible concepts, to describe an approach which could ensure that the objectives of the recent adopted legislation are met [33]. In our proposal, standardized real-world data (RWD) collection and standardized RWP-monitoring, alongside the interpretation of these into RWE [34] would be enabled at a platform level, facilitating the RWP-evaluation of health AOPs. The platform would consist of modules, each provided with underlying APIs/developer toolkits: (i) for the collection of patient-reported RWD; (ii) for the collection of clinician-reported RWD; (iii) for the prespecified analysis of RWD to generate RWE; (iv) for bringing together RWD for AOPs with the same intended purpose, clinical indication, and target population to analyze them together; and (v) for the display and sharing of trend data for each class of AOP to stakeholders.

With sequential development, the platform approach could deliver a set of performance measures including patient-reported outcome measures (PROMs) and patient-reported experience measures (PREMs), clinician reported outcomes (ClinROs) and clinician-reported experience measures (CREMs) as well as other RWD, e.g., data from EHR or billing data (Table 2). PROMs/PREMs would be delivered as in-app questionnaires to patients, whereas ClinROs/CREMs would be delivered as questionnaires to HCPs via the EHR. Information accessible through questionnaires could be derived from 3 types of questions: (i) general high-level AOP questions relating to metrics relevant to all AOPs; (ii) more specific questions subdivided by AOP disease, indication area or therapy type; and (iii) questions specific to the individual AOPs and its individual indication or claimed benefits and unique characteristics (Table 2).

**Table 2:** Examples of relevant performance measures included in the performance measurement system. These are just a few examples and do not cover all disease-specific outcome measures that will be collected.

Completed by	PROMs/PREMs and ClinROs/CREMs			other RWD
	High-level AOP questions	Disease/ indication/ therapy-specific AOP questions	Individual AOP-specific questions	
***Patient***	- MARS [35] - SUS [36] - EQ-5D-5L [37] - QOLS [38] - WHODAS 2.0 [50]	- CORE-OM [51] (mental health conditions) - PAID [40] (diabetes) - CHF-PROM [41] (chronic heart failure)	Targeted questions, e.g., on app usability or on certain app features	- Key performance indicators (e.g., average frequency of use, duration of use, drop-out rates, tap rate, etc.) - Routine data (e.g., from registers, hospital databases, or billing data) - Data from EHR (e.g., lab data, vital signs, medical images) - Physiological data (e.g., blood glucose levels, tracking of nutritional intake) - Health economic measures (e.g., QALY [52])
***HCP***	- MARS [35] - SUS [36]	- Rating scales, e.g., HAMD [43] - Performance- based ratings, e.g., Ashworth scale [42] - Testing of disease symptoms	Targeted questions, e.g., about quality of medical content	

MARS, Mobile App Rating Scale; SUS, System Usability Scale; QOLS, Quality Of Life Scale; WHODAS, WHO Disability Assessment Schedule; CORE-OM, Clinical Outcome in Routine Evaluation–Outcome Measure; PAID, Problem Areas in Diabetes Survey; CHF-PROM, Chronic Heart Failure-Patient Reported Outcome Measure; HAMD, Hamilton Depression Rating Scale; EHR, Electronic Health Record; QALY, Quality-Adjusted Life-Year.

For (i) general high-level AOP questions, there are already standardized questionnaire instruments or frameworks for PREMs/CREMs including the Mobile App Rating Scale (MARS) [35] and the System Usability Scale (SUS) [36] for assessing the engagement, functionality, usability, and information quality as well as the effectiveness, the efficiency and the satisfaction with which the patients and HCPs complete their tasks and achieve their goals. Outcome measures include generic PROMs, such as EQ-5D-5L [37] to measure the general health status of patients, or the Quality Of Life Scale (QOLS) [38], which is intended for patients with chronic diseases to determine the impact of healthcare when cure is not possible. Disease-specific questions (ii) are comprised of validated PROMs applicable to main AOP application areas including conditions affecting mental health (e.g., depression [39]), metabolic disease (e.g., diabetes [40]) and circulation (e.g., chronic heart failure [41]). ClinROs would include disease-specific rating scales [42,43] and testing of specific disease symptoms. Questions of category (iii) would be specific to the individual AOP and consist of questions on specific AOP features or on special medical content. Therefore, the design of the questionnaires would involve a combination of highly standardized options, as well as flexible options. The design of the latter would involve cooperation between the AOP developer and the assessor.

Some AOPs already use PROMs, either directly as part of their intervention to receive personalized information or instructions and thus to support patients’ health-related knowledge and actions or for evaluation to demonstrate their positive impact on healthcare [44]. However, some AOP developers do not use standardized PROMs and therefore do not ensure comparability with other AOPs. In contrast, we ensure a standardized outcome measurement with validated PROMs/PREMs. We do not propose to add yet more PROM/ClinRO questionnaires to these apps, and thus increase patient and HCP burden, but to use, whenever possible, the PROM/ClinRO questionnaires that are already included. It is particularly important to ensure that the interface design of the AOP is not disrupted and that the frequency of the questionnaires is proportionate to the purpose of the application so as not to limit the therapeutic effectiveness of the AOP. Simple user interfaces and clear instructions help to minimize the effort and expenditure of time for data entry and increase participation. Especially for busy HCPs, the seamless integration with the EHR can facilitate easier data entry, increasing the likelihood of HCP participation. It can also help to raise awareness among patients and HCPs about the added value that participation can have for the improvement of health apps and thus indirectly influence their own or their patients’ health improvement, respectively. Where it is claimed by the developer that an AOP will have a positive healthcare effect, then, on a population level, the effectiveness of the app should be demonstratable through showing improvement on a PROM (provided the impact can be measured using a PROM). If there are a number of AOPs used for the same clinical indication, target population and intended purpose (as there are currently are in the DiGA directory [45]), then comparisons of their effect on changing PROMs measured at defined time points after onboarding of the AOP and after the completion of the treatment process, as well as a short monthly questionnaire to monitor the course of treatment, would allow global comparison of AOP effectiveness. Automated reminders and notifications can help patients and clinicians to input data at the defined time points.

Digital tools, including AOPs, are exceptionally suited for gathering RWD, as numerous elements of a product’s meta-data can be collected effortlessly without requiring additional effort from patients or providers. Therefore, the proposed platform could collect the average frequency of use, the duration of use as well as the drop-out rates of the individual AOP. Additionally, routine data, like billing or claims data or data from registers or databases could be collected as well. Although claims and billing data were initially collected primarily for payment purposes, they can also be used to analyze patients’ and prescribers’ behaviors and interactions, e.g., to understand disease progression, to monitor AOP and medication usage, and to validate and replicate findings from clinical trials [46]. Outcomes that were reported by patients to their HCPs directly and were recorded in the EHR could also be collected through the platform if the patient provided its informed consent to this linkage [47]. In later stages of platform development, we envisage that it would be possible to link this data to outcomes through automated natural language processing (NLP)-based analysis of outcome data routinely recorded in the EHR [48,49]. NLP can be used to process texts and clinical notes in EHRs and transform them into structured RWD [48,49]. Furthermore, data indicating a pattern of frequent use at a long duration may indicate people enjoy using the app but does not necessarily correspond to health improvements. In the longer term, it is possible that depersonalized physiological data related to outcomes could be monitored (e.g., blood glucose levels for diabetes management AOPs or tracking of nutritional intake for obesity management AOPs). The monitoring and analysis of depersonalized streams of such data, collected through the platform, would ensure that the AOP actually fulfills its intended purpose, and would facilitate reimbursement decisions on objective data.

Our proposed approach for platform-based RWP-monitoring and evaluation will require further investigation and ongoing post-development optimization. A centralized monitoring platform for AOPs would need to be developed sequentially, starting as a simple concept, collecting PROMs/PREMs and ClinROs/CREMs as well as AOPs meta-data (e.g., average frequency of use), since these can be relatively easily collected via questionnaires or directly from the AOP (Table 2). The platform develops over time to include objective outcomes like depersonalized physiological data and data from EHRs as well as health economic data, like QALY (Table 2). The range of standardized instruments and their customization to AOP assessment is increasing over time, and this would be enabled by an extensible library of instruments, as are currently delivered through catalogs, including on clinical trial electronic data capture systems [53–55]. Since adaptability based on feedback is a core aspect of digital health applications, including AOPs, the pre-market and post-market phase of these applications are highly linked (much more than for non-digital medical devices) and they therefore require ongoing evidence gathering and evaluation processes, linked to approval and release and monitoring that account for their on-market changes and continuous iterative adaptive development (and intended improvement) [56]. The proposed platform could be used to gather data from the clinical investigations (i.e., clinical trials) that must be carried out for the post-market clinical follow-up of medical devices [56,57]. Post-market surveillance/RWD data could be collected within the proposed platform, which would have the great advantage of having the data available in bundled form for these related trials and data monitoring processes. As a future goal, the platform could be further adapted so that it can also be used to assist data collection for drug approval clinical trials, where AOPs or other software as a medical device are used within the trial protocol [58].

The data gathered and integrated through the approach would be in 2 phases. The first phase would consist of the delivery of questionnaires to patients and clinicians for ongoing assessment, and the linking and integration of these with data provided by the manufacturer on frequency and duration of use by questionnaire respondents, as suggested in recent draft proposals [33]. Assessment data collection for return to the centralized platform would be delivered in the AOP interface to the patient, for PREMs and PROMs [59,60] as well as via the EHR interface to the HCP, for CREMs and ClinROs [61,62].

This standardized approach could be delivered via a modular but single platform application programming interface (API), provided either directly from the responsible overseeing ministry or state agency (e.g., BfArM in Germany, FAMHP in Belgium, CPAM in France), or from a contracted private provider, with input from all stakeholders including AOP developers. Avoiding fragmentation, the data collection approach is necessary to ensure the utility of the data for comparative approaches. Key to the standardized approach would be the use of highly standardized sets of source questions to create the questionnaire instruments, transparent, prespecified constraints on their combination and prespecified approaches for data interpretation. Although such an approach could start on a voluntary basis, in the medium term, such a system would work best under regulatory rules which required the developer to build and provide their RWP-evaluation pipeline from their AOP to the platform, as part of the evaluation process of their app for the on-prescription program.

It is necessary that the proposed platform will be carefully designed based on user needs (patients, HCPs, data analysts) [63]. To ensure interoperability, the platform will need to integrate with various health data sources, like EHRs, wearables and mobile apps, using standardized interoperability protocols like Health Level 7 (HL7) or Fast Healthcare Interoperability Resources (FHIR). A user-friendly interface with customizable dashboards and visualization will make complex data understandable and ensure that the platform can be adapted to different user groups (patients, HCPs, data analysists) [63]. The platform approach we propose would require continuous improvement through regular updates, with new features and improvements based on user feedback and technological advancements. The implementation of the proposed performance monitoring platform would include developing a secure data collection framework, integrating app performance analytics, establishing compliance with health regulations, conducting pilot studies, and launching phased rollouts, with milestones such as initial framework setup, beta testing completion, regulatory approval, and full market deployment (as is state-of-the-art in software platform development [56]). Approaching platform development through step-by-step implementation makes it possible to react quickly to user feedback and to ensure the ongoing improvement of the platform as well as the alignment with stakeholder needs.

The use of standardized outcome measures offers many advantages, such as better visibility not only of comparative app performance but also maximizing digital inclusion. The potential benefits of DIGA may remain inaccessible to user groups that have less access to digital health technologies due to barriers to such as limited digital skills, low health literacy, or language challenges [64,65]. These factors can decrease the likelihood of adopting and consistently using digital tools, especially in health contexts. The outcome measurements by our proposed platform would enable tracking these barriers and therefore benefitting the equity of digital health systems by ensuring this technology reaches all segments of the population and providing insights into reasons for low engagement. However, this could also lead to app developers not wanting to implement their AOP into certain populations if there is a perceived risk of a decrease in their performance scores. In order to avoid this, performance metrics that account for diverse populations could be implemented in the platform. For example, the Quadruple Aim Framework emphasizes the interconnected nature of improved healthcare outcomes for the individual patient, the caregiver, population health, and reduced care costs, and has elsewhere been recommended as a way to standardize measurement of remote patient monitoring systems [66]. Metrics should be adjusted or weighted to reflect the different challenges and baseline health conditions of various demographic groups. This would ensure that AOPs are not unfairly penalized for working with more complex or high-need populations, and can therefore increase the chances of AOPs eventually demonstrating its impact on not just individual health but public health. Additionally, goals and benchmarks specific to different populations could be established. By comparing the AOPs performance within a particular demographic rather than across the general population, developers would be encouraged to address the unique needs of those groups without fearing negative impacts on their overall scores. In addition, as with all monitoring approaches, care needs to be taken to avoid system gaming. The specification of standardized endpoints could mean that AOP developers limit themselves to optimizing only these, in order to make their product more profitable. This problem can be addressed through the inclusion of a range of different outcome measures in the platform, including both more subjective PROMs and ClinROs and more objective RWD like data from EHR and physiological data as well as health economic measures like QALY [52] (Table 2).

### Data privacy, security, and regulatory challenges

There is ongoing discussion regarding the legal status of RWD collection through surveys for the purposes of safety, performance, and clinical benefit evaluation. Some interpretations are that this is only possible within the framework of a formal clinical investigation (implying the need to comply with all applicable legal and other requirements), and others interpreting that this could (depending on specifics), come under less stringent requirements of active post-market surveillance activities [67]. Legislative clarification may resolve this issue definitively, but either interpretation is compatible with our proposed framework. We propose that the systematizing of post-market evaluation of AOPs, through a single, centralized and platform-supported registry, delivered in partnership with the regulator, and following all clinical investigation requirements, is better than multiple fragmented studies, each of which would have to be notified to the regulator and independently designed and delivered by each AOP developer.

Regulatory compliance, patient consent, and ethical considerations should be taken into account to ensure compliant RWD collection through surveys for evaluating the safety, performance, and clinical benefits of AOPs. Implementing validated survey instruments in AOPs’ user interfaces requires careful consideration of data protection. The continuous assessment of AOP performance must prioritize user privacy, particularly for patients, and comply with the standards of the General Data Protection Regulation (GDPR). The GDPR sets stringent requirements for data collection, emphasizing the need for explicit patient consent and the lawful processing of health data [68]. Thorough investigations into data protection issues follow established GDPR-compliant root cause analysis procedures. Obtaining informed consent from patients is crucial [47,69]. Surveys and other data collection methods must ensure that patients are fully aware of how their data will be used, stored, and protected. Therefore, consent processes should be clear and comprehensive, covering the scope of data use, potential risks, and the benefits of participation. Informed consent ensures that data collection adheres to ethical standards and respects patient autonomy. It is fundamental for upholding patients’ rights and maintaining trust in the healthcare system. The quality and reliability of the data collected also tend to be higher, as patients are more likely to provide accurate and comprehensive information when they are fully informed [70]. It also can enhance patient engagement and empowerment by involving them in their own care and the evaluation of health technologies that may benefit them directly. Although the process of obtaining informed consent can be administratively burdensome and resource-intensive, this is not the case due to the digital application of the platform, as consent can be realized, for example, by integrating or forwarding it to consent management platforms [47]. Additionally, the questionnaire data collected within our proposed platform would be aggregated and presented to protect patient anonymity. These aggregated data also allow for more comprehensive analysis and better statistical power, leading to more reliable and valid results. It enables the identification of trends and patterns that individual data points may not reveal.

### Implementation chances and challenges

The described AOP performance assessment platform is technically feasible, through utilizing secure and trusted technological identity management methods for data storage, and state-of-the-art approaches in secure cloud computing [71]. These interfaces establish secure connections to external applications, ensuring safe execution of tasks on AOP systems and secure sharing of structured data through standardized authentication protocols. This description of the platform concept does not focus on technical implementation, but a simple analogy to the networked approaches most citizens adopt to the handling of sensitive data in their daily lives, e.g., in mobile banking, suggests that data security technical challenges would not preclude such a platform, if there was will and resource to develop it, following industry best practice.

For AOP developers, the effort required to bring a new AOP to market is already substantial (Table 1), and additional requirements are to be introduced in the next few years [5]. In the course of the development of an AOP, development costs, production costs, study costs, certification costs, quality assurance costs must be incurred. In order for there to be private-developed AOPs, these must be remunerated sufficiently, in the short, medium, and long term. Common sense would seem to dictate that increasing requirements for RWD and RWE delivery will mean that developers must use ever more resources to be and stay approved as AOPs. We propose that the introduction of a platform concept for RWD, may partially increase AOP developer workload, but it would also reduce their workload, particularly for technical, or administrative regulatory work as well as costs. A platform approach for centralizing and unifying RWD assessment and evaluation would avoid the need for each developer to source, setup, and run their own independent RWD-gathering initiatives, which is not a trivial task. Instead, the developer’s technical workload would be shifted to building their AOP’s interface to the platform, and their individual data connection pipeline, in cooperation with the AOP assessing body. Where requirements would be undoubtedly greater are in the optimization of each AOP’s product performance to deliver a positive impact on healthcare. As sectoral trend plots would show AOP performance of all comparable AOPs (e.g., AOPs in the same disease category) in standardized metrics, the developers of poorly performing AOPs would be under immediate pressure to improve performance through delivering better AOP products [72,73]. Even market leaders would see the need for continuous improvement to stay in their leading position and to be the top recommendation of clinicians and choice of patients and clinicians. This race to the top would cost money, but for improving apps, and could be supported through pricing that fairly rewards costs. There is of course the risk that metrics could be “gamed,” but shared approaches, transparency, and ongoing evaluation of the delivery of the platform, as well of the individual AOPs is likely to lead to more trustworthy assessment of care delivery effect. System gaming, where developers manipulate metrics to present artificially improved performance, poses significant risks, such as misleading stakeholders, compromising patient care, and undermining the credibility of the platform. Fairness and robustness against system gaming is likely to be much greater with this proposed system, than for the alternative of individual RWP-monitoring solutions built and delivered by each developer in isolation. Implementing stringent auditing mechanisms, regular checks, and anomaly detection algorithms can further mitigate the risk of manipulation. Additionally, fostering a culture of accountability and ethical development practices among developers, combined with regular independent reviews, can ensure that the performance measurements remain accurate and reliable. By addressing these risks comprehensively, the platform can maintain its integrity and continue to provide valuable insights into the effectiveness of digital health apps.

However, the delivery of any joined up-platform approach is challenging, and centralized, public sector software platform projects often run into delays and technical challenges [74], even when tendered to private companies. Technical challenges could include the heterogeneity of data sources, since RWD comes from various sources such as EHRs, mobile health apps, and wearable devices, and in some cases, data harmonization techniques will be required. Compliance with standards like HL7 and FHIR is essential but can be challenging to achieve across diverse data systems. Ensuring secure and compliant data sharing practices between different stakeholders (HCP, patients, AOP developers) is essential to maintain trust and legal compliance. Additionally, protecting the platform from cyber attacks, data breaches, and other security threats is paramount. This involves implementing robust encryption, access controls, and continuous monitoring for vulnerabilities. We therefore propose a step-by-step development and integration of the platform based on user feedback to resolve technical problems and other issues at an early stage. Some positive examples of platform projects have demonstrated successful delivery at high speed, particularly during the COVID-19 pandemic [75].

## Conclusion

In order to ensure that performance-based remuneration can be based upon reliable and transparent RWD, we propose a system for ongoing AOP performance assessment. Anonymized data collected from standardized patient and HCP experience and outcome measures would be transmitted to the regulator to support price negotiations. There would be the possibility of extension of this concept, in later phases, with evaluation of EHR data for consenting patients, to integrate PROMs/CROMs data with direct measurement of clinical outcome measures. The system proposed would reduce developers’ administrative workload for RWD-gathering and evaluation, as the standard platform delivers a service that developers must only interface with, instead of build themselves. The platform would create “a race to the top” as relative performance of comparable AOPs would transparently be made available. This would be in the interest of patients, doctors, and healthcare systems, but reimbursement needs and duration of contract terms needs to justify the investment of AOP developers. Although costs and challenges to building an infrastructure for standardized RWD data collection and monitoring for digital health will be high, it avoids the paradoxical situation of digital solutions being innovative in their development, and digitally connected in their care delivery, but lacking truly digital performance monitoring. There is a large need to enable price-setting bodies, payers and patients to monitor solutions so that they are not in the dark as to comparative performance. When new hardware is installed in safety-critical settings, they are designed with standardized conduits for information flow, so that their performance can be monitored and underperforming components can be replaced. AOPs must also be monitorable, to those who prescribe them, pay for them, and use them.

AOP companies might bridle against complete transparency of their AOP performance, but we argue that this conservative thinking, inspired through protection of commercial positions and intellectual property, is in reality highly limiting to the commercial success of the sector. There is a great opportunity for the whole AOP sector in terms of quality improvement and consequently greater acceptance among HCPs and patients. Functioning participatory and transparent feedback loops would lead to greater AOP optimization, more enthusiastic adoption by patients and, in turn, this would lead to the growth of the entire AOP sector to the benefit of all. As the saying goes, “a rising tide lifts all ships,” but in the healthcare sector, not all AOPs “ships” should be passively lifted, but rather those that funnel part of their reimbursement to their ongoing maintenance and seaworthiness. Here, we have the opportunity, through short-term investment in achievable infrastructure, and through bravery of the sector in adopting transparency, to develop truly effective and transformational AOP programs.
